# Epilepsy life skill education guidelines for primary school teachers and learners in Limpopo and Mpumalanga Provinces, South Africa: Multiphase mixed methods protocol

**DOI:** 10.1371/journal.pone.0271805

**Published:** 2022-07-22

**Authors:** Thendo Gertie Makhado, Rachel Tsakani Lebese, Maria Sonto Maputle, Lufuno Makhado

**Affiliations:** 1 Faculty of Health Sciences, Department of Advance Nursing Sciences, University of Venda, Thohoyandou, South Africa; 2 Faculty of Health Sciences, Research Office, University of Venda, Thohoyandou, South Africa; 3 Faculty of Health Sciences, Department of Public Health, University of Venda, Thohoyandou, South Africa; Wachemo University, ETHIOPIA

## Abstract

Epilepsy is a disorder in which nerve cell activity in the brain is disturbed, causing seizures. It may result from a genetic condition and occurs mainly in children, especially at a primary level. Most people living with Epilepsy suffer from stigma and discrimination because of a lack of knowledge regarding Epilepsy. This study aims to develop life skills education guidelines for primary school learners of Limpopo and Mpumalanga provinces to educate learners about Epilepsy, thus decreasing stigma and discrimination. A multimethod research approach will be used in this study to fulfil its purpose. Both stages 1 and 2 of the empirical phase (phase 1) will employ an exploratory-descriptive study design focusing on the primary school teachers, life skills educational advisors and learners to obtain their perceptions or views regarding the need to include Epilepsy in life skills education. Data will be collected using individual interviews for life skill educational advisors and focus group discussions for teachers and learners at the sampled primary schools in Limpopo and Mpumalanga provinces. Phase two will conceptualise the phase 1 findings into the conceptual framework, and phase 3 will develop and validate the life skills guideline. This study will adhere to both internal and external ethical considerations. Recommendations will be made based on the findings of the study.

## Introduction

Epilepsy is regarded as a brain disease that is very common and affects about 70 million people worldwide, and it is characterised by the presence of seizures [1]. Seizures associated with Epilepsy may last for a few minutes, characterised by a sudden fall, blank stare, or jerking movements. According to Magazi et al. [2], there are two types of epilepsy seizures: motor and non-motor seizures. Motor seizures include tonic, myoclonic, clonic, tonic-clonic, atonic, hyperkinetic seizures, automatisms, and epileptic spasms. Moreover, non-motor seizures include absence, cognitive and emotional, sensory, and autonomic seizures [2]. Adequate knowledge related to Epilepsy is of great importance and a necessity worldwide because misconceptions and stigma surround this condition.

A study conducted in western countries indicates some of the misconceptions attached to the condition, which describes Epilepsy as a supernatural condition because the word ’epilepsy’ in Greek means *"being seized by forces from without*" [3]. This is also supported by Dolo et al. [4] in their study conducted in Congo, which also explained that Epilepsy is the condition caused by evil spirits, saliva, or touching same-sex individuals during seizures. These misconceptions may negatively affect people living with Epilepsy (PLWE) because they feel stigmatised and discriminated against, leading to fear and anxiety [5,6]. Furthermore, there is a perception that PLWE are not supposed to get married and are excluded in social activities, resulting in self-blame and shame [7]. These misconceptions are caused by several factors identified by Musekwa et al. [8], who revealed that poor education and low socioeconomic status contribute to the misconceptions and stigma toward Epilepsy. In support, Yeni et al. [6], and Kartal and Akyildiz [9] also emphasised that insufficient knowledge and cultural beliefs about Epilepsy contribute to misconceptions. The aftermath of the identified misconceptions and stigma toward Epilepsy can potentially impact the health-seeking behaviours of PLWE.

World Health Organization (WHO) [10] reported that PLWE might not seek treatment/health care because of fear of being identified with the disease due to stigma and misconception related to the condition. Furthermore, some reports strengthening epilepsy education might increase health-seeking behaviour. Not being educated or having poor knowledge about a phenomenon can negatively impact an individual’s life.

Poor education can lead to poor health, unemployment, poor health-seeking behaviour, depression, exploitation, etc. [3,7,8]. Poor education regarding Epilepsy impacts the lives of PLWE, parents and caregivers to PLWE and community members. This was affirmed by O’Neill et al. [7], who revealed that due to lack of education regarding Epilepsy, many children dropped out of school or were denied access to education because of the perception that a learner with Epilepsy can contaminate other learners at school. Similarly, Herman et al. [3] have explained that a lack of epilepsy education can lead to ignorance about treatment and social exclusion by the community. Evidently, poor education regarding Epilepsy is found in the community and among other stakeholders and a serious issue among school children and teachers.

Studies show that Epilepsy is the most common disorder in childhood, indicating that the prevalence of Epilepsy is much higher from the age of 4–10 [11,12]. Additionally, about 0.7% of school-age children have Epilepsy which makes it possible that every school has a child that has Epilepsy [11,12]. When a child at school has an epileptic attack in the classroom, it can affect other learners and disturb the classroom. Therefore, knowledge regarding Epilepsy is of paramount importance among teachers and learners. According to Owolabi et al. [13], teachers lacked adequate knowledge regarding Epilepsy, resulting in the separation of learners with Epilepsy from those without Epilepsy. Moreover, Whiting-MacKinnon and Roberts [14] revealed that learners with Epilepsy felt isolated as they were treated differently from other learners and teased by other learners. This predisposes the affected learners from receiving support, care, and assistance during an epileptic seizure, let alone the preventive measures.

Studies published worldwide indicate that epilepsy education receives less focus than it does on other chronic diseases [15–17]. Therefore, there is poor education regarding Epilepsy worldwide [18,19].

The South African curriculum on life skills and life orientation focuses less on Epilepsy, as indicated in Table 1, where only in grade 9 students get to learn about Epilepsy. This is serious given the need for awareness among school children in terms of Epilepsy, its signs and symptoms, behavioural and necessary steps one takes in case of an episode of seizure or epileptic attack. Based on Grade, the following Life Skills and Life Orientation topics are covered as reflected in (Table 1).

**Table 1:** Life skill and life orientation focus per grade.

Grade 1	Healthy Habits	
Grade 2	Healthy living	
Grade 3	Health Protection and healthy eating	
Grade 4–6	No Health-related issues included	
Grade 7	No Health-related issues included	
Grade 8	No Health-related issues included	
Grade 9	Common diseases: tuberculosis, diabetes, Epilepsy, obesity, anorexia, HIV and AIDS Causes of diseases: social, economic and environmental factors, including use of alcohol and tobacco, poor eating habits and physical inactivity Treatment options, care and support Resources on health information and health services Strategies for living with tuberculosis, diabetes, Epilepsy, HIV and AIDS	Epilepsy covered
Grade 10	Development of the self in society Social and environmental responsibility	
Grade 11	Development of the self in society Social and environmental responsibility	
Grade 12	Development of the self in society Social and environmental responsibility	

Although, there had been studies conducted in South Africa indicating some guidelines aimed at helping teachers on how to be able to recognise and manage learners with Epilepsy [20–23]. Little is known about the availability of appropriate life skill guidelines that focus on school learners themselves in terms of their ability to recognise and manage fellow learners should an epileptic attack present itself when none of the teachers are available to offer help. It should be noted that school-age and adolescent age is regarded as the most important ages when intellectual capabilities are very high. However, there is scarce research regarding education to learners regarding Epilepsy. It is, therefore, of paramount importance for learners in primary levels to be furnished with life skill education focused on Epilepsy as they play an essential role in the lives of fellow learners, family members, and community members.

There seems to be neglect in the provision of epilepsy life skill education towards rural-based primary school learners in Limpopo and Mpumalanga provinces. According to Makhado et al. [25], it was emphasised that *"a holistic understanding of Epilepsy is key given that it has been found and understood to exist within two parallel worlds*: *the first is based on the scientific advances in the management and treatment of Epilepsy where enormous scientific progress has been witnessed; the other concerns a religious and traditional world characterised by beliefs*, *superstitions and prejudice related to Epilepsy that remain quite resistant to the numerous Western initiatives for people living with Epilepsy (PLWE)*. *Strong traditional values*, *practices and beliefs contribute to delays in the presentation and diagnosis of Epilepsy in the available healthcare systems of people in the rural communities of South Africa*.*"* Thus, the Department of Basic Education (DoBE) lacks involvement in bridging the identified gap through life skills education. The latter is evident given the focus of most guidelines, policies and programmes found in South Africa mainly aimed at providing such education to learners living with Epilepsy [20–23]. This is a huge problem given that fellow learners living without Epilepsy need to be aware, conscious, and in a capacity to recognise the onset of seizures as well as any potentially visible signs and symptoms of Epilepsy, reporting and taking necessary steps to assist where possible during any form of an episode. The identified problem can increase stigma and discrimination towards learners living with Epilepsy and encourage misconceptions and maltreatment of such learners. Within the issues, the need to develop epilepsy life skills guidelines for primary learners to help curb the misconceptions, stigma and discrimination, and maltreatment of learners living with Epilepsy while promoting timely response to the onset of epileptic signs and symptoms, referral and proper support thereof.

### Conceptual framework

**Fig 1:**
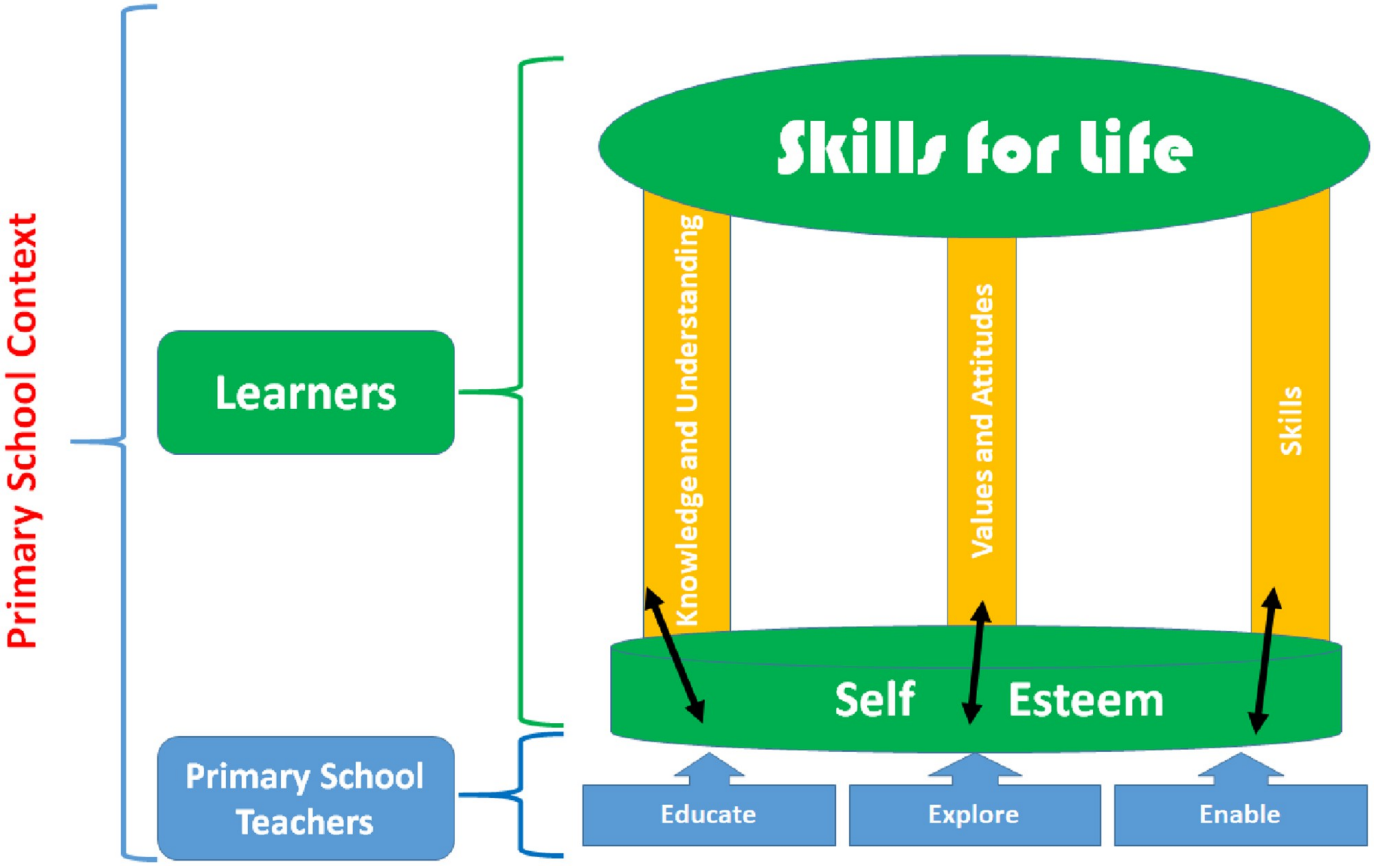
The life skill approach model adapted from the SNR Consultancy & Training (2010).

The three-legged stool model will guide this study, consisting of three pillar concepts and foundational support [24]. The interaction of the three pillars is represented graphically in Fig 1 as a three-legged stool [24]. According to the analogy of a three-legged stool, all three legs must be present and structurally sound at the same length [24]. This three-legged stool model is applicable in the life skills approach, wherein each leg should be as strong as the others, and all need to be a firm foundation of support. As a result, the three legs of this study are composed of three pillar concepts for the skills for life, which are:

- Knowledge and Understanding
- Values and Attitudes
- Skills

#### Knowledge and understanding of Epilepsy

Given the lack of knowledge and understanding of Epilepsy in the public, which is manifested through increased misconceptions [8,25–33], the primary school learners will need to know and understand Epilepsy from the education provided in the primary schools.

#### Values and attitudes towards Epilepsy

The link between knowledge of and attitudes and traditional solid values toward Epilepsy has been documented by various studies [6,25,34,35]. Thus, positive attitudes toward Epilepsy had been predicted by adequate knowledge and understanding [6,36–40]. Therefore, it is imperative that a thorough exploration of the values and attitudes towards Epilepsy, positive values and attitudes can be instigated on primary school learners. Primary school learners will be facilitated in exploring values and attitudes toward Epilepsy to establish what is positive and negative to stimulate appropriate values and attitudes for a better understanding of PLWE.

#### Skills related to Epilepsy

According to Braga et al. [32], children require to be trained and educated in small groups through an enabling approach. This has the potential for children to improve their knowledge about Epilepsy, their competency and skills related to dealing with seizures when a fellow student is having an episode and their social interactions with PLWE [32].

These legs, as aforementioned, should have a firm foundation (individual’s self-esteem) which is constantly strengthened through the three core elements provided by the primary school teachers, which include:

- Educating- Promotes knowledge and understanding of Epilepsy
- Exploring- Promotes positive values and attitudes toward Epilepsy
- Enabling- Promotes skills toward Epilepsy (as well as proper social interaction)

The interconnectedness of the three-legged stool and its foundational support is presented in Fig 1.

## Materials and methods

### Study aim

This study aims to develop life skills education guidelines for primary school learners of Limpopo and Mpumalanga.

### Study objectives

The objectives of this study are provided under the different phases.

### Phase 1: Empirical phase

- To explore the perceptions/views of primary school teachers and life skills educational advisors regarding the need to include Epilepsy in life skills education. *(Stage 1*: *Qualitative study)*
- To explore the perceptions of primary school learners regarding the need to include Epilepsy in life skills education *(Stage 2*: *Qualitative Study)*

### Phase 2: Conceptualising the findings into the conceptual framework

- To conceptualise the empirical phase findings into the conceptual framework that will guide the development of epilepsy life skills guidelines.

### Phase 3: Development of Epilepsy life skills guidelines for primary learners

- To develop Epilepsy life skills guidelines for primary learners in Mpumalanga and Limpopo Provinces.
- To test and validate the developed Epilepsy life skills guidelines

### Study design and setting

A research approach is a plan that guides the research process step by step. It outlines the critical steps involved in the whole research and how they will be integrated. The study is going to be conducted in three phases. The first phase would gather the empirical data from respondents. The data would be analysed and then informed the second phase, which would involve conceptualising the findings into the conceptual framework that would guide the third phase, which involves developing and validating the epilepsy life skills education guidelines for primary school learners Limpopo and Mpumalanga. The empirical phase will employ a qualitative multimethod approach, which will utilise two independent, yet interdependent research methods to complement one another and address the study’s primary aim. Therefore, the researcher will use two qualitative approaches (referred to hereafter as stages).

**Fig 2:**
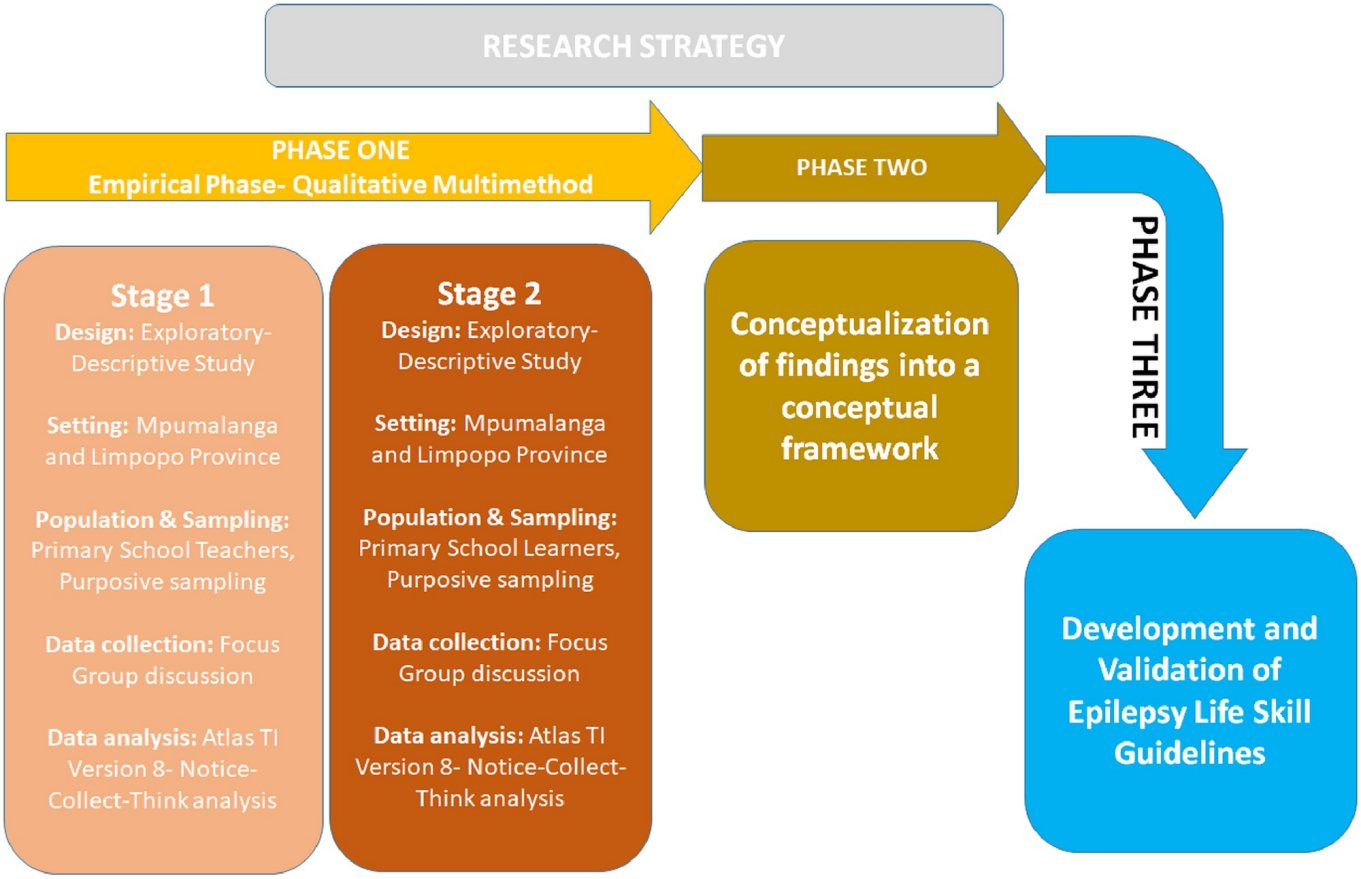
Illustration of the Study’s research approach.

Stage 1 of the empirical phase will be an exploratory-descriptive study focusing on the teachers’ perceptions or views regarding the need to include Epilepsy in life skills education and possible key epilepsy life skills education content that can be included in primary education level from Grade to Grade. This will be followed by stage 2 of the empirical phase, which will also be an exploratory-descriptive study focusing on primary learners’ perceptions regarding the need to include Epilepsy in life skills education and the important life skills elements of Epilepsy that Primary learners would like to learn. The results of the two studies will be conceptualised into the conceptual framework (Phase 2) that will be used to guide the development and validation of the epilepsy life skill guidelines for primary school learners (Phase 3) for Mpumalanga and Limpopo Province. See Fig 2 for the research approach illustration.

#### Phase One: Empirical Phase- Stage 1 and 2 a qualitative multimethod design

Multimethod research is used in this study to fulfil its purpose. Multimethod research uses various methodologies and methods of research and qualitative and quantitative techniques [41]. Multimethod research is characterised by the co-existence of different research methods [42]. Furthermore, multimethod studies are not limited to qualitative and quantitative methods but can combine different methods to answer various questions in one study; Clark and Ivankova [42,43] discuss how researchers use a variety of qualitative and quantitative methods to conduct multimethod research. According to Cresswell [44], the distinction between mixed-method and multimethod is that mixed-method research is the collection analysis and integration of quantitative and qualitative methods. In contrast, multimethod involves collecting and analysing multiple types of data, whether qualitative, quantitative or both [44].

Briefly, multimethod research comprises several different research methods to answer one research question or parts of it in a manner that complements the others. A qualitative multimethod approach is proposed in this study. It will utilise two independent yet interdependent research methods to complement one another and address the study’s main aim. Therefore, the researchers will use two approaches (referred to hereafter as stages), both in qualitative methods. The multimethod design will promote the exploration of primary school teachers and life skills advisors’ influence on knowledge and understanding of Epilepsy and the values and attitudes towards Epilepsy among primary school learners. Promoting values and attitudes toward Epilepsy and promoting knowledge and understanding of Epilepsy are the strong pillars of the three-legged stool model that is guiding the current study. This will be carried out in the following manner:

#### Stage 1: Qualitative study

This first stage of the proposed study will be an exploratory-descriptive study exploring the perceptions of primary school teachers and life skills educational advisors regarding the need to include Epilepsy in life skills education. According to Polit and Beck [45], exploratory-descriptive studies are used to shed light on how a phenomenon manifests and can be particularly useful for uncovering the true nature of little known phenomena. In addition, exploratory-descriptive research has been described as a non-traditional study in which a naturalistic inquiry is conducted to gain insight into a phenomenon. In these types of studies, the variables under study are not controlled, and the data are used to develop a theory or explain the phenomenon from the participants’ perspective [46,47].

#### Stage 2: Qualitative study

This second stage of the proposed study will be an exploratory-descriptive study exploring primary school learners’ perceptions regarding the need to include Epilepsy in life skills education.

### Setting of the study

This study will be conducted in two South African provinces, namely Limpopo and Mpumalanga. The province of Limpopo is in the far north of South Africa, bordering Zimbabwe, Botswana, and Mozambique, while Mpumalanga is in the east of South Africa, bordering Swaziland and Mozambique. There is a proximity between the two provinces. The researchers chose to concentrate their attention on these two provinces since they contain various population groups. The two provinces are also geographically and culturally connected, accommodating the most diverse cultures from other South African provinces.

Limpopo and Mpumalanga provinces are populated and characterised by diverse, multicultural groups of people. Thus, mostly Basotho, Pedi, Zulu, Swathi, Ndebele, Venda and Tsonga are within Limpopo province, while Mpumalanga province has a vast mixture of Tsonga, Ndebele and Swati cultures. Limpopo has been reported to be regarded as the fifth largest population province in South Africa, with an overall population of 4.9 million people. In comparison, Mpumalanga is ranked the 6th most populated province in South Africa, with an overall population of 4.7 people [48]. There has been an increase in access to basic services in Limpopo and Mpumalanga provinces. However, there are still disparities between urban, peri-urban and rural areas. The two provinces are characterised by dense rural areas that are underserved in healthcare provision. Mpumalanga province’s unemployment rate is 35.2%, while Limpopo province is 30.4% [48]. Using this information, the researcher determined it as a population of interest and will extract the study sample from this population.

### Study population

For the first stage of the study, school teachers and life skills educational advisors will be the target population for possible inclusion in the study. Teachers and life skills educational advisors are the people who help learners acquire knowledge and competence, so they can tell if the inclusion of Epilepsy in life skills education for primary students is necessary. For the second stage of this study, primary school learners will be the target population. Primary school learners in Limpopo and Mpumalanga who are in grade 4 to grade 7 will be targeted for possible inclusion in the study.

### Sampling methods and sample size determination

Sampling is the process of selecting a group of people, events, behaviours or other elements that represent the population being studied [49]. According to Brink et al. [50], non-probability is one of the types of sampling which allows the researcher to judge and select participants who know the most about the phenomenon. Grove and Gray [51] said that in non-probability, not every participant could participate in the study. Purposive sampling is a judgmental or selective technique of sampling in which the researcher consciously selects certain participants, elements, events or incidents to include in the study [51].

#### Sampling of the provinces and rural communities

Purposive sampling was used to sample the villages based on cultural diversity. The study is part of the GladAfrica Epilepsy Research Project (GERP), thus operating within the already selected rural communities Limpopo and Mpumalanga provinces. For Limpopo province, the selected rural communities include Malavuwe/Nweli, Mtititi, Bochum and Modjadjiskloof. For Mpumalanga province, the selected rural communities include Clara, Acornhoek, Jerusalem and Kwaggasfontein.

#### Sampling of the Schools

Total population sampling will be used to sample all primary schools located within the selected rural communities of Limpopo and Mpumalanga provinces of South Africa.

#### Sampling for primary school teachers and life skills educational advisors

This study will employ a non-probability purposive sampling method to purposely select primary school teachers and life skills educational advisors to participate in the study. It is with the judgement that the primary school teachers and life skills educational advisors have a better understanding of the need for epilepsy life skills education. The researchers intend to conduct six focus group discussions at the primary schools in selected rural communities in Mpumalanga and Limpopo. The number of participants in each focus group will depend on the number of teachers available at school who meet the inclusion criteria. However, the target will be five to eight participants per focus group at a time, and the number of sessions will depend on data saturation. The researchers will sample only teachers and life skills educational advisors that meet the criteria of being a teacher and having a qualification that enables them to be recognised as a teacher in terms of the Employment of Educators Act, 1998 (Act 76 of 1998) and who teaches or has taught life skills and has at least 12 months of experience facilitating a life skill class in Limpopo and Mpumalanga province. Furthermore, life skills educational advisors working within the designated circuits within which the selected schools are located.

#### Sampling of primary school learners

This study will employ a non-probability purposive sampling method to purposely select primary school learners from grade 4 to grade 7 to participate in the study with the judgement that these groups of primary school learners at this stage have a better understanding of the need for education on Epilepsy. Sampling techniques have already been defined in stage 1. The researchers intends to conduct a focus group discussion at the Primary schools selected rural communities in Mpumalanga and Limpopo province. We intend to conduct six focus groups, thus, three in each province. The number of participants in each focus group will depend on the number of learners available at school who meet the inclusion criteria. However, the target will be 8 to 12 participants per focus group at a time, and the number of sessions will depend on data saturation. It is believed that learners participate more when they are in groups; hence FGD will be used to collect data. We will sample only learners who meet the criteria of being primary school learners in grade 4 to grade 7 selected communities of Limpopo and Mpumalanga province.

### Data collection

#### Primary school teachers (Stage 1)

The researchers will use focus group discussions (FGD) to collect data among Primary School Teachers. The researchers opted for the focus group discussions among teachers because this data collection method is ideal when you want to elicit various perspectives on a defined topic [52]. The researchers seek to gather perceptions/views of primary school teachers regarding the need to include Epilepsy in life skills education and the possible key content that can be included. The focus groups will be guided by semi-structured FGD guides (Annexure E) to facilitate the discussion, and two main central questions are:

- *What are your perceptions/views regarding the need to include Epilepsy in life skills education*?
- *What is the possible key epilepsy life skills education content that can be included in primary level from Grade to Grade*?

All the focus group discussions will be audio-recorded with permission from the participating teachers and life skills educational advisors. Continuous collection of the field notes will be maintained throughout the FGD.

#### Educational subject advisors (Stage 1)

Data will be collected through in-depth semi-structured interviews among life skills educational advisors. Individual semi-structured interviews are chosen given the number of life skills educational advisors, which is very low and would not be ideal for FGD. In the study, the researcher seeks to gather perceptions/views of personal life skills educational advisors regarding the need to include Epilepsy in life skills education and the kind of content that could be specifically included. The individual interviews will follow a semi-structured interview guide (Annexure D) focusing on the following central questions:

- *What are your perceptions/views regarding the need to include Epilepsy in life skills education*?
- *What is the possible key epilepsy life skills education content that can be included in the primary level from Grade to Grade*?

#### Primary school learners (Stage 2)

We will use focus group discussions (FGD) to collect data among Primary School learners. The researchers opted for the focus group discussions because this data collection method is ideal when you want to elicit various perspectives on a defined topic [52]. The researchers seek to gather perceptions/views of primary school learners regarding the need to include Epilepsy in life skills education and the possible key content that can be included. During the FGD, there will be an allocated break time and games to keep the learners interested in participating and refresh the learner’s memories. The focus groups will be guided by semi-structured FGD guides (Annexure F) to facilitate the discussion, and the two main central questions are:

- *What are your perceptions/views regarding the need to include Epilepsy in life skills education*?
- *What are the important life skills elements of Epilepsy that you would like to learn*?

All the focus group discussions will be audio-recorded with permission from the participating teachers. Continuous collection of the field notes will be maintained throughout the FGD.

### Data management plans

This study will use ATLAS.ti for data analysis and follow the basic notice-collect-think (NCT) analysis steps. Furthermore, the basic steps will enable the researcher to work systematically instead of declaring the software to be the method itself [53]. We will start by noticing aspects of the data that lead to an idea for a label and begin to collect what is noticed in the form of codes [53]. This coding will be divided into descriptive-level and conceptual-level analyses.

#### Descriptive-level analysis

This level of analysis comprises two stages, namely first stage coding and second stage coding.

- **First-stage coding**

The researchers will start by reading the interview and FGD transcripts and field notes, followed by the noticed patterns of the data, then write the notes, mark segments and attach the first preliminary codes, which could either be descriptive or already conceptual [53]. This first coding phase ends when the researcher no longer notices anything new, when no codes can be added, and the researcher can only apply existing ones [53]. We will then look for code labels that have been used only a few times as these codes are more likely to be descriptive, referring to specific data segments, but without the ability to be connected. According to Friese [53], such codes are candidates for a closer examination, either to merge them with similar codes under a higher-order conceptual label or to evaluate whether they can be collected under a common category level. This process aims to develop subcategories and categories and develop them conceptually into themes [53].

- **Second-stage coding**

According to Friese [53], the second phase of coding serves as a way to validate the code list. If the code list is developed usefully, not many new codes can be added at this stage. Therefore, the data will be ready for the next level of analysis.

#### Conceptual-level analysis

According to Friese [53], at this stage, the researchers will then link data using the network views function, exploring the already developed ideas further from the first stage coding and integrating all findings in writing and graphical representations. Thus, categories and themes will be developed.

### Measures to ensure trustworthiness

Trustworthiness in qualitative research is achieved by enhancing Credibility, Dependability, Conformability, and transferability [54].

- **Credibility**-The credibility of the study will be enhanced by spending more time with participants in focus group discussions until data saturation is reached. The audio-recorded interviews will be transcribed, interpreted, and shared with participants to validate if their experiences are competently and accurately captured.
- **Dependability**-Dependability will be enhanced by maintaining an audit trail by keeping all copies of notes, and transcribed and recorded data for future use, including supplying participants with researchers’ personal and academic information for contact or explanation.
- **Confirmability-** To ensure confirmability, promoters will review the research study and will confirm that the researcher has reported correct information based on the data collected. The researcher will ensure that the study is confirmable by having another professional (independent co-coder) in their field analyse the data collected and compare the findings, which should yield similar results.
- **Transferability**- To ensure transferability, biographical information will be obtained; a dense description of the research methodology and the findings will be provided. Additionally, participants’ background information will be densely described to ensure transferability. The context and setting of the study will be described so that others may gauge the transferability of the findings.

### Phase 2: Conceptualisation of findings into a conceptual framework

This phase will present the conceptualisation of the phase 2 findings into the life skill approach model to come up with the conceptual framework that will guide the development of the epilepsy life skill guidelines.

### Phase 3: Development and validation of the Epilepsy life skill guidelines

The development and validation of the epilepsy life skill guidelines will adapt the World Health Organization’s globally accepted guidelines development model [55] and shall follow the following process:

- Establish the guidelines topic
- Provide/justify the need for the guidelines
- Review existing life skills guidelines
- Form the epilepsy life skills guidelines steering group (includes *primary school teachers*, *life skills educational advisors*, *school governing body representatives*, *health care professionals and health promotion officers*)
- Establish the scope of the epilepsy life skills guidelines
- Declaration and conflict of interest among potential Steering group members
- Formulate key epilepsy life skills guidelines questions in PICO format
- Perform systematic reviews of the evidence for each key question formulated
- Evaluate the quality of the evidence for each important outcome, using the Grading of Recommendations, Assessment, Development and Evaluations (GRADE) framework as appropriate
- Formulate recommendations using the GRADE framework
- Draft the epilepsy life skills guidelines document
- Conduct external peer review (Validation)
- Finalise the epilepsy life skills guidelines document
- Review and approve the final epilepsy life skills guidelines

### Ethics approval and consent to participate

Ethical clearance was sought from the University of Venda’s Ethics Clearance Committee (**SHS/19/PH/37/2101**). Permission to conduct the study was also sought from the Provincial Department of Education of Mpumalanga and Limpopo provinces. Permission to conduct the study in the specific study sites within each province was sought from traditional leaders (Traditional chiefs and headmen). Written consent was sought from all participants. Since data would also be collected from primary school children (under the age of 18), written "consent" would be sought from their parents/legal guardians, and primary school children themselves would "assent" to be part of the study. Information sheets and assent forms would be provided for participants to read and sign if they agree to be part of the study.

The study protocol had been approved by the University of Venda Higher Degree Committee and had received ethical clearance (**SHS/19/PH/37/2101**). The authors are in the process of community entry in both provinces to obtain access to the study participants and start with data collection.

## Discussion

To the best of our knowledge, epilepsy life skills guidelines for primary school learners have not been developed anywhere globally. Education regarding Epilepsy is very prominent because it has the potential to decrease stigma and misconceptions regarding Epilepsy. Kaddumukasa et al. [56], Hermann et al. [3] and Musekwa et al. [8], in their studies, also supported that when epilepsy education is strengthened, there is a reduction of stigma and misconceptions around it. Hashemi et al. [57] also emphasised that it is of great importance when most people are knowledgeable about Epilepsy because, through the knowledge, they develop skills and are able to know how to manage an individual with an epileptic attack.

The current study aims to develop an epilepsy life skills guideline for primary learners in Limpopo and Mpumalanga Province. It is believed that equipping individuals with knowledge regarding a specific phenomenon may build a positive attitude towards that phenomenon, especially at a younger age. Therefore it is believed that when learners are educated about Epilepsy at a younger age may reduce stigma and misconception about Epilepsy. Epilepsy life skills education will also help PLWE to have good health-seeking behaviour [58–60]. This is supported by Lewis et al. [61], who reported that increasing knowledge effectively improves self-care. Molla et al. [62] emphasised that poor understanding and unfavourable knowledge contribute to poor health-seeking behaviour. This means that it is essential to educate learners at a younger age to promote positive attitudes and values toward Epilepsy, thus decreasing misconceptions and stigma. Furthermore, through Epilepsy education, living skills with PLWE will be empowered, helping people to know how to react when seizures occur and treat PLWE.

The study’s limitations include using a qualitative approach that diminishes the generalizability of the findings to the broader population. We will be using non-probability purposive sampling to sample participants for the study. However, we have made efforts to include all primary schools within the selected study setting through total population sampling for all the primary schools to be included in the study, although the chance of being sampled in each school is not equal. Any amendments to the study will be done through the University Higher Degree Committee and the Human and Clinical Trial Research Ethics Committee. Only after approval, the amendment will be effected in the study.

## Conclusions

Our planned study will expand our understanding of the Epilepsy life skills needs in rural communities of Limpopo and Mpumalanga provinces. With this understanding, the development of Epilepsy lifeskills guidelines for primary school learners will be achieved to increase and improve the knowledge and awareness of Epilepsy. Thus, in turn, curb the development of Epilepsy related misconceptions, myths, stigma and discrimination. Given the poor awareness and knowledge or understanding of Epilepsy in rural communities, the development of Epilepsy life skills guidelines will emerge as a vital tool to promote awareness and understanding of Epilepsy. The findings will have significant implications on the epilepsy educational life skills provision and the community in general.

## Supporting information

S1 File (ZIP)
