# Supplementary material for: Epilepsy life skill education guidelines for primary school teachers and learners in Limpopo and Mpumalanga Provinces, South Africa: Multiphase mixed methods protocol
Source: PLoS One. 2022 Jul 22;17(7):e0271805. doi: 10.1371/journal.pone.0271805 (PMC9307198; doi:10.1371/journal.pone.0271805)
Supplement: S1 File — (ZIP) [file pone.0271805.s001.zip › INTERVIEW GUIDE 2022.docx]

**INTERVIEW GUIDE AND FOCUS GROUP DISCUSSION GUIDE**

# **INTERVIEW GUIDE FOR LIFE SKILLS EDUCATIONAL ADVISORS AND PRIMARY SCHOOL TEACHERS**

**INTRODUCTION**

Thank you for being here and agreeing to participate in this study. This study aims to develop life skills education guidelines for primary school learners of Limpopo and Mpumalanga as explained in the consent form. Now we need to find out from your point of view if it is important to include epilepsy in the life skills education for primary learners and to find out what can be included in the life skills guideline. You are not going to be called by your real name in this interview, you will be referred to as participant ‘A, B, C’ and so on to protect your identity and your answers will be kept confidential.

**QUESTIONS**

1. What are your perceptions regarding the need for including epilepsy in life skills education?
2. What is the possible key epilepsy life skills education content that can be included in primary level from grade to grade?

# **FOCUS GROUP DISCUSSION FOR LEARNERS**

**INTRODUCTION**

**ESTABLISHING RAPPORT BETWEEN THE RESEARCHER AND LEARNERS**

Thank you for being here and agreeing to participate in this study. This study aims to develop life skills education guidelines for primary school learners of Limpopo and Mpumalanga as explained In the consent form. Now we need to find out from your point of view if it is important to include epilepsy in the life skills education for primary learners and to find out which elements of epilepsy you think you would like to learn. Remember to call yourselves ‘A’ then the next one will be ‘B’, the third one will be ‘C’ and so on until we all have a letter that is assigned to us as a code that we will be called by for this discussion.

**QUESTIONS**

1. What are your perceptions as learners regarding the need for including epilepsy in life skills education?

BREAK: PUZZLE GAME

1. What are the important life skills elements of Epilepsy that Primary learners would like to learn?

BREAK: WORDS GAME
