# Supplementary material for: Epilepsy life skill education guidelines for primary school teachers and learners in Limpopo and Mpumalanga Provinces, South Africa: Multiphase mixed methods protocol
Source: PLoS One. 2022 Jul 22;17(7):e0271805. doi: 10.1371/journal.pone.0271805 (PMC9307198; doi:10.1371/journal.pone.0271805)
Supplement: S1 File — (ZIP) [file pone.0271805.s001.zip › HCTREC Ethics certificate.PDF]

RESEARCH AND INNOVATION
OFFICE OF THE DIRECTOR

NAME OF RESEARCHER/INVESTIGATOR:
Prof L Makhado

STAFF NO:
13663

PROJECT TITLE: Epilepsy intervention programme in rural communities of Limpopo and Mpumalanga provinces.

PROJECT NO: SHS/19/PH/37/2101

SUPERVISORS/ CO-RESEARCHERS/ CO-INVESTIGATORS

NAME	INSTITUTION & DEPARTMENT	ROLE
Prof L Makhado	University of Venda	Investigator – Staff
Dr A Maphula	University of Venda	Investigator
Dr JT Mabunda	University of Venda	Co- Investigator
Prof SM Maputle	University of Venda	Co- Investigator
Prof RT Lebese	University of Venda	Co- Investigator
Snr. Prof LB Khoza	University of Venda	Co- Investigator
Mrs TG Makhado	University of Venda	Co- Investigator
Mr MJ Chueng	University of Venda	Co- Investigator
Ms M Nemathaga	University of Venda	Co- Investigator
Ms Q Chabangu	University of Venda	Co- Investigator

Type: **Staff Research**
Risk: **Minimal risk to humans, animals or environment**
Approval Period: **January 2020 – December 2023**

The Human and Clinical Trails Research Ethics Committee hereby approves your project as indicated above.

General Conditions

While this ethics approval is subject to all declarations, undertakings and agreements incorporated and signed in the application form, please note the following.

- The project leader (principle investigator) must report in the prescribed format to the REC:
  - Annually (or as otherwise requested) on the progress of the project, and upon completion of the project
  - Within 48hrs in case of any adverse event (or any matter that interrupts sound ethical principles) during the course of the project.
  - Annually a number of projects may be randomly selected for an external audit.
- The approval applies strictly to the protocol as stipulated in the application form. Would any changes to the protocol be deemed necessary during the course of the project. The project leader must apply for approval of these changes at the REC. Would there be deviated from the project protocol without the necessary approval of such changes, the ethics approval is immediately and automatically forfeited.
- The date of approval indicates the first date that the project may be started. Would the project have to continue after the expiry date; a new application must be made to the REC and new approval received before or on the expiry date.
- In the interest of ethical responsibility, the RECs retains the right to:
  - Request access to any information or data at any time during the course or after completion of the project,
  - To ask further questions; Seek additional information; Require further modification or monitor the conduct of your research or the informed consent process.
  - withdraw or postpone approval if:
  - Any unethical principles or practices of the project are revealed or suspected.
  - It becomes apparent that any relevant information was withheld from the REC or that information has been false or misrepresented.
  - The required annual report and reporting of adverse events was not done timely and accurately,
  - New institutional rules, national legislation or international conventions deem it necessary

ISSUED BY:
UNIVERSITY OF VENDA, RESEARCH ETHICS COMMITTEE
Date Considered: January 2020

Name of the Chairperson of the Committee: Dr NS Mashau

Signature: [signature]

UNIVERSITY OF VENDA
OFFICE OF THE DIRECTOR
RESEARCH AND INNOVATION
2021 -11- 29
Private Bag X5050
Thohoyandou 0950

University of Venda
PRIVATE BAG X5050, THOHOYANDOU, 0950), LIMPOPO PROVINCE), SOUTH AFRICA
TELEPHONE (015) 962 8504/8313 FAX (015) 962 9060
"A quality driven financially sustainable, rural-based Comprehensive University"
